# Severely Impaired Learning and Altered Neuronal Morphology in Mice Lacking NMDA Receptors in Medium Spiny Neurons

**DOI:** 10.1371/journal.pone.0028168

**Published:** 2011-11-21

**Authors:** Lisa R. Beutler, Kiara C. Eldred, Albert Quintana, C. Dirk Keene, Shannon E. Rose, Nadia Postupna, Thomas J. Montine, Richard D. Palmiter

**Affiliations:** 1 Department of Genome Sciences, University of Washington, Seattle, Washington, United States of America; 2 Howard Hughes Medical Institute and Department of Biochemistry, University of Washington, Seattle, Washington, United States of America; 3 Department of Pathology, University of Washington, Seattle, Washington, United States of America; Case Western Reserve University, United States of America

## Abstract

The striatum is composed predominantly of medium spiny neurons (MSNs) that integrate excitatory, glutamatergic inputs from the cortex and thalamus, and modulatory dopaminergic inputs from the ventral midbrain to influence behavior. Glutamatergic activation of AMPA, NMDA, and metabotropic receptors on MSNs is important for striatal development and function, but the roles of each of these receptor classes remain incompletely understood. Signaling through NMDA-type glutamate receptors (NMDARs) in the striatum has been implicated in various motor and appetitive learning paradigms. In addition, signaling through NMDARs influences neuronal morphology, which could underlie their role in mediating learned behaviors. To study the role of NMDARs on MSNs in learning and in morphological development, we generated mice lacking the essential NR1 subunit, encoded by the *Grin1* gene, selectively in MSNs. Although these knockout mice appear normal and display normal 24-hour locomotion, they have severe deficits in motor learning, operant conditioning and active avoidance. In addition, the MSNs from these knockout mice have smaller cell bodies and decreased dendritic length compared to littermate controls. We conclude that NMDAR signaling in MSNs is critical for normal MSN morphology and many forms of learning.

## Introduction

Sensory and motor information processed by the cortex and thalamus passes through the striatum where it is modulated by two largely antagonistic classes of MSNs that express distinct dopamine receptors and neuropeptides [1]. The activity of the two classes of MSNs is modulated by dopaminergic input from the ventral midbrain as well as various populations of striatal interneurons [2], [3]. Both classes of MSNs send GABAergic projections to brain regions outside the striatum, which ultimately project back onto the thalamus and cortex. Through its modulation of cortical and thalamic input, and via downstream neural circuitry, the striatum contributes to the generation of goal-directed behavior. Thus, disruptions of dopamine signaling, interneuron function, or MSN integrity by disease processes or intentional manipulation of laboratory animals, impair learning and cognition [4]–[7].

The excitatory, glutamatergic input onto MSNs activates AMPA-type glutamate receptors, NMDARs and metabotropic glutamate receptors [8]. Studies of each of these receptor sub-classes in the striatum has revealed their importance for striatal function [9], [10]; however, the precise role of each of these receptor types in various forms of learning remains incompletely understood. Throughout the brain, NMDARs are thought to be particularly important in learning due to their long-lasting open times [11], calcium permeability [12], and facilitation of long-term potentiation (LTP) [13]. Both direct and indirect evidence implicates NMDARs in the striatum in several types of learning [14]–[20]. In addition to their role in transmitting glutamate signals in mature animals during learning, NMDARs have been implicated in neuronal development in several brain regions [21]–[23].

NMDARs are tetramers that require two essential NR1 subunits for assembly of a functional receptor [24]. Mice with a conditional allele of the unique gene *Grin1*, which encodes the NR1 subunit, have been crossed to mice expressing Cre recombinase selectively in the striatum. The results of these studies have confirmed that NMDAR currents are absent in neurons lacking NR1 and that LTP cannot be elicited in striatal slice preparations from these animals [18], [25]. However, the behavioral consequences differ in these studies, perhaps due to incomplete knockout of striatal NR1 protein in the mice that were less severely affected [18], or expression of Cre recombinase in striatal interneurons as well as MSNs [26], [27]. We have generated a conditional *Grin1* knockout that selectively and completely depletes NMDARs from both populations of MSNs, while leaving those in interneurons intact. These mice have significantly smaller MSNs with shorter dendrites than littermate control mice. Although they are grossly normal, these knockout mice are completely incapable of several forms of learning.

## Results

### Generation of knockout mice

**Figure 1:**
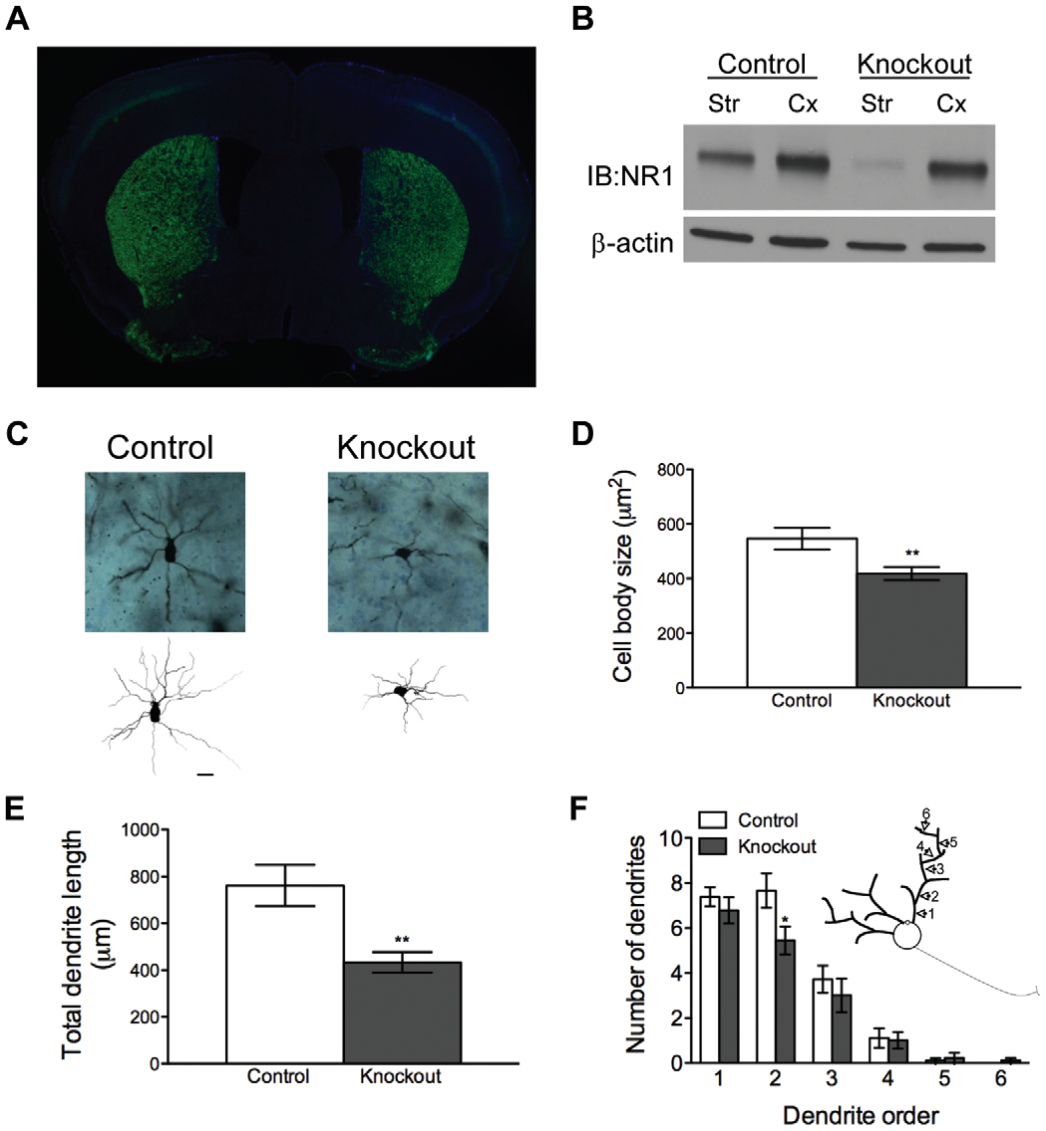
Knockout mice that selectively lack NMDAR in striatal MSNs have abnormal MSN morphology. *(A*) GPR88-CreGFP is expressed selectively in the striatum of knockout animals. A DAPI counterstain was performed. (*B*) NR1 Western blot of striatal (str) and cortical (cx) homogenates from control and knockout animals. (*C)* Representative micrographs and tracings of MSNs from control and knockout animals (20 µm scale bar). (*D)* Cell body size of MSNs in control and knockout mice (n = 18 neurons per genotype); *P* = 0.009 by unpaired t test; **P<0.01. (*E)* Total MSN dendrite length in control and knockout mice (n = 18 neurons per genotype); *P* = 0.002 by unpaired t test; **P<0.01. (*F)* Number of dendrites by dendrite order in control and knockout mice (n = 18 neurons per genotype); *P<0.05 by unpaired t test. Diagram in (*F*) depicts the definition of dendrite order.

Functional NMDARs were removed from all MSNs by inactivation of *Grin1* specifically from these neurons. Mice with a floxed *Grin1* locus (*Grin1* ^lox/lox^) were crossed to animals in which Cre recombinase expression is driven by the endogenous *Gpr88* locus (*Gpr88^CreGFP/+^*) and heterozygous at the *Grin1* locus (*Grin1^Δ/+^*) to generate mice with the genotype *Gpr88^CreGFP/+^*; *Grin1^Δ/lox^* (referred to as knockout mice) and littermates with the genotype *Gpr88^CreGFP/+^*;*Grin1^lox/+^* (referred to as control mice). It has been reported that GPR88 expression is primarily restricted to MSNs within the striatum [28]. In agreement with this finding, GFP fluorescence was restricted primarily to nuclei of cells in the striatum of *Gpr88* ^CreGFP/+^ mice, although low levels of expression in the cortex were observed (Fig. 1*A*). However, although NR1, which is essential for the formation of functional NMDARs [24], was dramatically reduced in the striatum of knockout mice, NR1 levels in the cortex were normal (Fig. 1*B*). Residual NR1 could be contributed by non-MSNs in the striatum. Coupled with repeated demonstrations from our lab and others that NR1 expression is required for NMDAR signaling [18], [24], [25], [29], these findings confirm that these knockout mice lack functional NMDAR signaling selectively in the striatum.

### MSN morphology is abnormal in knockout mice

Because signaling through NMDARs is thought to be important for neuronal development [21]–[23], we predicted that MSNs in knockout mice might manifest abnormal morphology. Striatal tissue from 3 control and 3 knockout mice was processed for Golgi staining and imaging (Fig. 1*C*). Analysis revealed that MSN cell bodies from knockout mice were significantly smaller than those in control animals (Fig. 1*D*, all statistics are reported in figure legends), and total dendritic length was significantly shorter in knockout mice (Fig 1*E*). Control and knockout animals had similar dendritic branching patterns, but knockout animals trended toward having fewer branches of each dendrite order. This deficit was significant in second-order dendrites (Fig 1*F*).

### Knockout mice are smaller than normal but exhibit normal baseline locomotion

**Figure 2:**
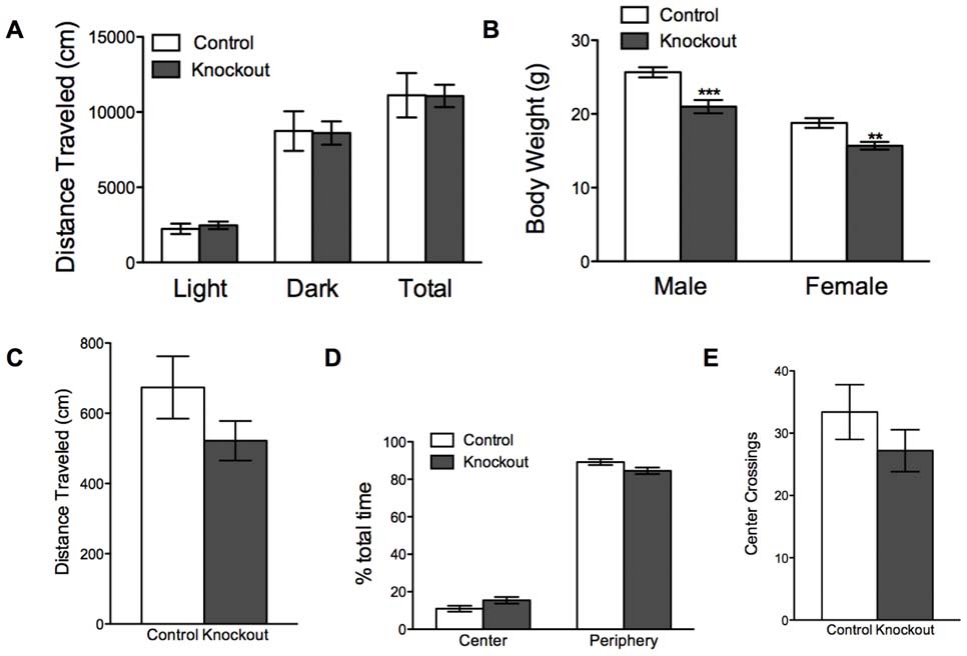
Knockout mice are smaller than normal but have normal spontaneous locomotion, and intact exploratory behavior. *(A)* Spontaneous locomotion by control (n = 10) and knockout (n = 11) animals; light cycle, *P* = 0.85; dark cycle, *P* = 0.93; total, *P* = 0.98 by unpaired t tests. (*B*). Body weight of 8-week-old animals; control (n = 15) and knockout (n = 12) males, *P*<0.0001; control (n = 11) and knockout (n = 10) females, *P* = 0.002 by unpaired t tests; **P<0.01; ***P<0.0001. (*C*) Locomotor activity of control (n = 10) and knockout (n = 5) animals in an open field; *P* = 0.17 by unpaired t test. (*D*) Fraction of time spent in the center and periphery of the open field by control (n = 10) and knockout (n = 5) animals; *P* = 0.08 by unpaired t tests. (*E*) Number of open-field center crossings by control (n = 10) and knockout (n = 5) animals; *P* = 0.28 by unpaired t test.

Locomotor behavior of knockout and control mice was monitored for 24 hr. Knockout and control animals exhibited comparable locomotion during both the light and dark phases of the light cycle (Fig. 2*A*). In addition, both male and female knockout mice had significantly lower body weights than littermate controls at 8 weeks of age, the time point at which behavioral testing began (Fig. 2*B*). Beyond their slightly smaller size, knockout mice were not obviously distinguishable from littermate control animals.

### Knockout mice exhibit open field activity comparable to littermate controls

Knockout and control mice were placed in an open field for 10 min, and their total locomotion and exploratory behavior were monitored. Knockout mice traveled a distance comparable to control mice during this test (Fig. 2*C*). Furthermore, there was no difference between genotypes in the amount of time spent in the center of the open field (Fig. *2*D), nor was there a difference in the number of center crossings exhibited by either group (Fig 2*E*). These results indicate that lack of NMDAR signaling in MSNs does not hamper exploratory behavior. The normal amount of time spent in the center of the field suggests that the knockout mice are not anxious.

### Knockout mice exhibit impaired motor coordination and learning

**Figure 3:**
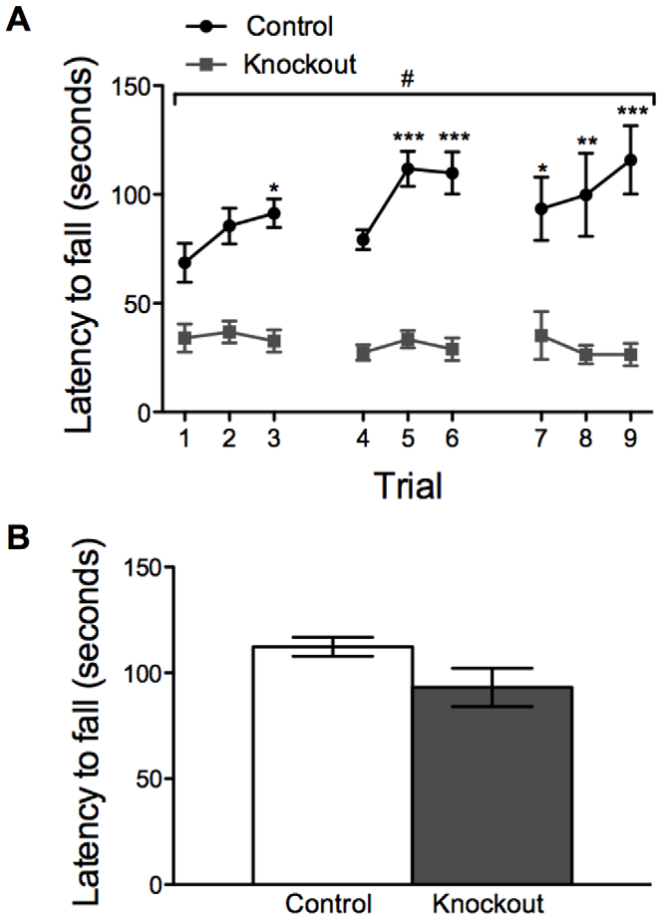
Knockout mice exhibit impaired locomotor learning. (*A*). Rotarod performance by control (n = 12) and knockout (n = 11) animals across days; two-way, repeated-measures ANOVA: genotype effect *F*(1, 21) = 70.5, *P*<0.001; day effect *F*(8, 168) = 1.63, *P* = 0.12; genotype x day effect *F*(8, 168) = 2.37, *P* = 0.02 (#*P*<0.05); *P<0.05, **P<0.01, **P<0.001 compared to trial 1 within genotype. (*B*) Latency to fall during a wire-grip test in control (n = 12) and knockout (n = 11) animals; *P* = 0.08 by unpaired t test.

Knockout and control mice were tested on an accelerating rotarod three times a day for three days to determine whether knockout mice have impairments in motor learning. While control mice learned robustly, as indicated by increasing latency to fall, knockout mice failed to improve across trials (Fig. 3*A*). Furthermore, knockout mice appeared to have impaired baseline performance on the rotarod, suggesting that these animals may also have a deficit in motor coordination. To explore whether these differences might be due to decreased strength or muscle tone rather than a motor-learning deficit, we tested grip strength in the same cohort of knockout and control mice by measuring latency to fall from an inverted wire grid. There was no difference in latency to fall between groups (Fig. 3*B*). This finding agrees with previous observations in a similar knockout mouse strain [18] and underscores the importance of NMDAR signaling in MSNs in motor learning.

### Knockout mice exhibit impaired learning in an operant-conditioning task

**Figure 4:**
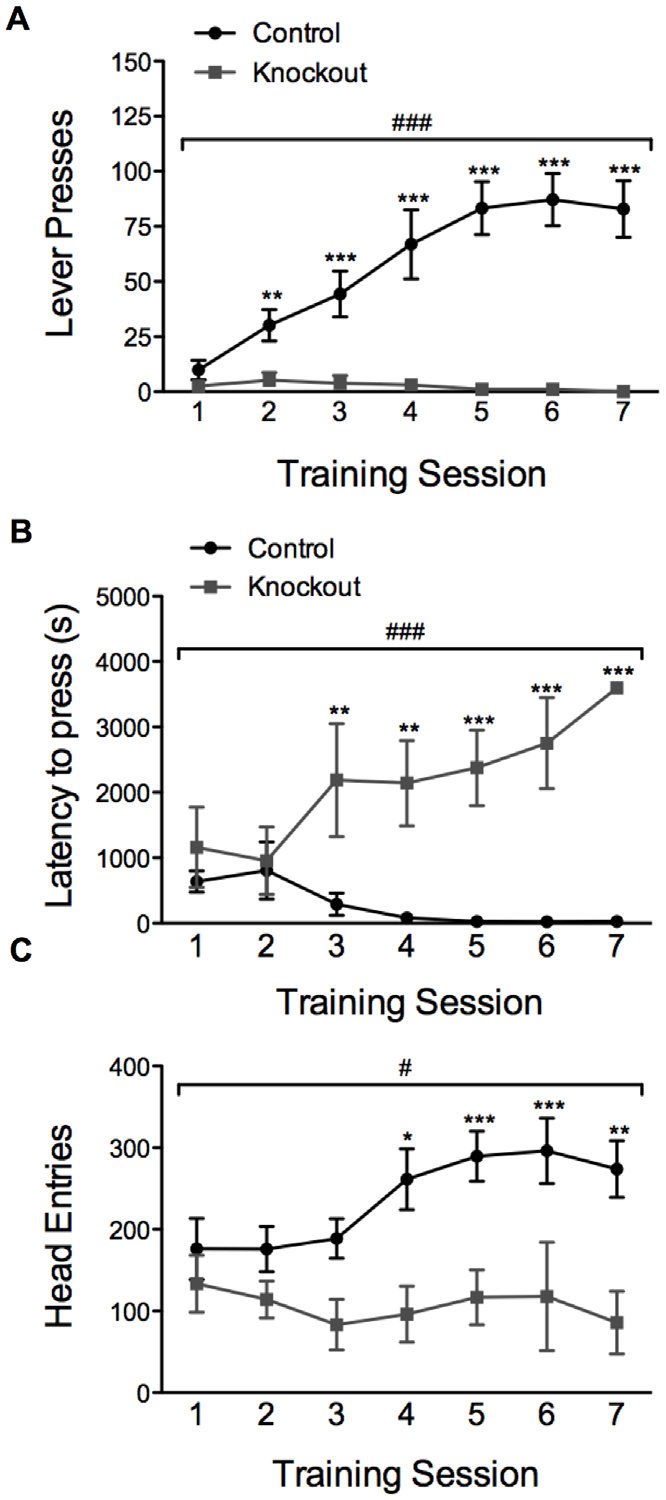
Knockout mice fail to learn a simple (FR1) operant task. (*A*) Lever presses during operant conditioning sessions by control (n = 10) and knockout (n = 5) animals across days; two-way, repeated-measures ANOVA: genotype effect *F*(1, 13) = 15.72, *P* = 0.002; day effect *F*(6, 78) = 10.90, *P*<0.001; genotype x day effect *F*(6,78) = 12.96, *P*<0.001 (###*P*<0.001); **P<0.01, ***P<0.001 compared to session 1 within genotype. (*B*) Latency to first lever press during operant conditioning sessions by control (n = 10) and knockout (n = 5) animals across days; two-way repeated measures ANOVA: genotype effect *F*(1, 13) = 38.75, *P*<0.001; day effect *F*(6, 78) = 2.26, *P* = 0.04; genotype x day effect *F*(6,78) = 8.04, *P*<0.001 (###*P*<0.001); **P<0.01, ***P<0.001 compared to session 1 within genotype. (*C*) Total number of head entries during operant conditioning sessions by control (n = 10) and knockout (n = 5) animals across days; two-way repeated measures ANOVA: genotype effect *F*(1, 13) = 10.14, *P* = 0.007; day effect *F*(6, 78) = 1.93, *P* = 0.08; genotype x day effect *F*(6,78) = 2.25, *P* = 0.04 (#*P*<0.05); *P<0.05, **P<0.01, ***P<0.001 compared to session 1 within genotype.

To determine whether knockout mice are capable of learning an appetitive operant-conditioning task, we tested the ability of knockout and control animals to learn a fixed ratio task in which one lever press leads to the delivery of one food pellet (FR1). Mice were given 60-min sessions on seven consecutive days during which the number of lever presses was measured. Control animals readily learned to lever press for food pellets, whereas knockout mice failed to do so (Fig. 4*A*). Considering the poor rotarod performance of the knockout mice, the impaired performance in this task might be due to motor deficits; however, this is an unlikely explanation for the severe operant conditioning impairment that we observed for a number of reasons. First, the latency to first lever press was similar in knockout and control animals at baseline, although the latency decreased in control mice across days, whereas it increased in knockout mice (Fig 4*B*). In addition, knockout and control animals had a similar number of head entries into the food receptacle at baseline; however head entries by control animals increased as they learned the task, whereas head entries by knockout animals did not change significantly (Fig 4*C*). Finally, the criterion for entering the training phase of the operant conditioning task was to eat all 10 pellets delivered in a 15-min magazine training session. Furthermore, although they earned fewer pellets than control mice, knockout mice retrieved and consumed all pellets during the training sessions, suggesting that these animals are coordinated enough to participate in this behavioral paradigm and that altered consummatory behavior is not the underlying cause of this behavioral deficit.

### Knockout mice exhibit impaired fear learning

**Figure 5:**
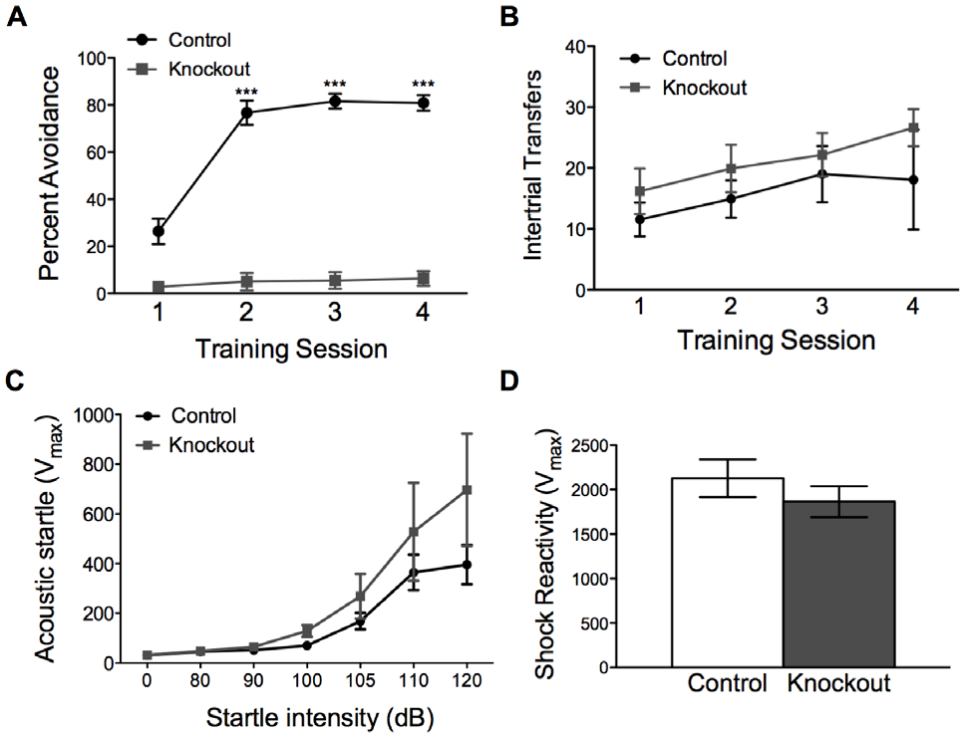
Knockout mice fail to learn two-way active avoidance. (*A)* Percent shock avoidance by control (n = 11) and knockout (n = 11) animals across training sessions; two-way, repeated-measures ANOVA: genotype effect, *F*(1, 3) = 165.6, *P*<0.001; time effect *F*(3, 60) = 97.0, *P*<0.001; genotype x time effect *F*(3, 60) = 76.4, *P*<0.001 (###*P*<0.001); ***P<0.001 compared to block 1 within genotype. (*B)* Intertrial transfers by control (n = 11) and knockout (n = 11) mice during two-way active avoidance sessions; two-way, repeated-measures ANOVA: genotype effect *F*(1, 20) = 1.18, *P* = 0.29; day effect *F*(3, 60) = 2.77, *P* = 0.05; genotype x day effect *F*(3, 60) = 0.26, *P* = 0.86. *(C*) Acoustic startle response curve for control (n = 8) and knockout (n = 6) mice across startle intensities; two-way, repeated-measures ANOVA; genotype effect *F*(1, 12) = 1.87, *P* = 0.20; startle intensity *F*(6, 72) = 18.03, *P*<0.001; genotype x startle intensity *F*(6, 72) = 1.25, *P* = 0.29. (*D)* Shock reactivity in control (n = 10) and knockout (n = 5), *P* = 0.35 by unpaired t test.

The two-way active avoidance paradigm tests both an animal’s ability to learn to associate a cue with a foot shock, and its ability to learn to engage in a behavior that prevents shock delivery. Two-way active avoidance was assessed in knockout and control mice as described [30]. Briefly, mice were placed in a two-compartment chamber with free access to both compartments. For each trial, a tone (7 s) predicted a foot shock, which the mouse could avoid by moving to the other compartment of the chamber during the tone presentation. Knockout and control mice were trained in this paradigm with 100 trials per day for four consecutive days, and their percent avoidance was scored by session. Control mice learned to avoid the foot shock, whereas knockout mice failed to do so (Fig. 5*A*). The behavioral difference in session 1 is likely due to intra-session learning by control animals. To assess whether impaired learning in this paradigm might be caused by reduced locomotor activity in the chambers, we analyzed the number of times knockout and control animals moved from one compartment of the chamber to the other during intertrial intervals (intertrial transfers). Both groups of animals had a slightly increased number of intertrial transfers across training sessions, but there was no difference in the number of intertrial transfers between knockout and control mice across days (Fig 5*B*). Failure to learn in this paradigm was not due to an inability to hear the cue, as knockout and control mice responded similarly to acoustic startle across a range of decibels (Fig. 5*C*). Because body weight might affect our assessment of acoustic startle, we tested whether normalizing to body weight might change our interpretation of these data. At the sound intensity used in the two-way active avoidance paradigm (80 dB), acoustic startle is not significantly different between groups when normalized to body weight (Vmax/body weight in controls: 2.2±0.4 vs knockouts: 2.8±0.7, *P* = 0.41 by unpaired *t* test). At higher decibel levels, the knockout animals appear to have a slightly increased startle response; however, this does not reach significance, even when the startle response is normalized to body weight (Vmax/body weight in controls: 17.7±3.4 vs knockouts: 35.4±8.5, *P* = 0.06 by unpaired *t* test). This enhancement of startle at highter dB levels may reflect increased fearfulness of the knockout animals, which warrants further study; however, this result suggests it is unlikely that the impaired two-way active avoidance learning in knockout animals is the result of an reduced acoustic startle response. Shock reactivity in knockout and control mice also was not significantly different, indicating that the failure of knockout animals to learn is not the result of impaired shock sensation (Fig. 5*D*). As with acoustic startle, normalizing this result to body weight did not change our interpretation of the data (Vmax/body weight in controls: 106.4±13.0 vs knockouts: 95.7±3.4, *P* = 0.56 by unpaired *t* test). Therefore, we conclude that animals lacking NR1 in MSNs did not learn how to avoid the foot shock based on the predictive tone.

## Discussion

We have generated a genetic mouse model in which Cre recombinase expressed at the *Gpr88* locus selectively ablates NR1 expression in all MSNs within the striatum. Others have shown that similar genetic models lack striatal NMDAR currents, and fail to elicit LTP in striatal slice preparations [18], [25]. These findings are in general agreement with a large amount of evidence implicating NMDAR-mediated calcium entry in facilitating LTP in many types of neurons [13], [31]. We have used this model to show that NMDARs on MSNs are required for normal MSN morphology in adult animals. They are not required for survival in our vivarium or for normal 24-hr spontaneous locomotion in mice. However, NMDARs on MSNs are critical for learning in each of the motor, fear, and appetitive tasks that we examined. In addition, NMDARs on MSNs are required for normal MSN morphology in adult animals.

The finding that striatal NMDARs are not required for survival or normal baseline functions including baseline locomotion and grip strength is consistent with data from a similar model in which Cre recombinase expressed from the striatum-specific *Rgs9* locus was used to inactivate the *Grin1* locus [18]. By contrast, a theoretically similar mouse model in which striatum-specific excision of *Grin1* was driven by a transgenic *Dlx5/6*-Cre line had a much more severe phenotype [25]. These discrepant findings may be due to a number of factors. It is possible that Cre-mediated recombination was more effective in the transgenic *Dlx5/6-Cre* line than in either of the knock-in strains; however, Western blot analysis in the knockout mice used here revealed near total absence of striatal NR1, with the small amount of residual NR1 most likely due to expression in interneurons. An alternate explanation is that the cell types affected in the transgenic line are different than those affected in either of the targeted Cre-expressing lines. Both RGS9 and GPR88 have been shown to be expressed in both dopamine D1- and D2-receptor-expressing MSN populations [26], [28]. RGS9 is also expressed in cholinergic interneurons in the striatum, whereas GPR88 is expressed exclusively in MSNs. *Dlx5/6*-Cre, on the other hand, is expressed in both classes of MSNs as well as all striatal interneuron types examined [27]. Expression in brain regions outside the striatum may also cause the behavioral differences. The knockout mice used in this study, for example, express CreGFP in a few cortical cells and in the inferior olive, which may explain some aspects of their phenotype. Another possible explanation for the phenotypic differences between these strains of mice is the age of onset of Cre recombinase expression in the three lines of mice. *Dlx5/6*-Cre is turned on at E12.5 [27]. Although developmental expression of *Rgs9* in mice has not been studied, it is first expressed in rats on E16 [32]. The timing of *Gpr88* expression in rodents is not known, and merits further study. Regardless, it is possible that the removal of NMDAR signaling at earlier or later times during development may have different effects on adult phenotype due to the role of NMDARs in synaptic development [33]. Finally, strain effects may underlie some of the phenotypic differences in these three mouse models.

Impaired learning on the rotarod by our knockout mice is also consistent with that reported for RGS9-Cre-mediated NMDAR knockout mouse [18]. Intact grip strength by the knockout mice suggests that impaired muscle tone is not the underlying cause of their poor rotarod performance. Furthermore, normal baseline locomotion and intact amphetamine sensitization [29] suggest that generally impaired locomotion is not responsible for this deficit. Our results are consistent with the well-characterized role of the striatum in maintaining locomotor coordination [4] and emphasize the importance of NMDAR signaling in mediating this type of learning.

The knockout mice also failed to learn a FR1 instrumental task. Both pharmacological [14] and genetic [34] evidence have implicated striatal NMDAR signaling in the acquisition of lever pressing for food rewards. Of note, in a previous genetic study, operant conditioning in *Rgs9-*Cre-mediated NMDAR knockout mice was present, although severely impaired [34]; by contrast, learning in the knockout mice used in this study was completely absent. Many of the same factors described above might explain this behavioral difference in two theoretically very similar mouse models. Specifically, the knockout mice used in this study have more extensive striatal NMDAR knockdown than the *Rgs9*-Cre-mediated NR1 knockout mice based on Western blot data presented in the two studies. Alternatively, the knockout mice used in our study do express Cre recombinase in cells outside the striatum, e.g. the inferior olive [28], which may account for the more severe instrumental conditioning deficits that we have observed. The ventral striatum, including the nucleus accumbens, has a well-established role in appetitive learning [35]. Pharmacological experiments indicate that NMDARs in the dorsal striatum [20] and nucleus accumbens [14] are important for this form of learning. Because our knockout mice lack NMDARs throughout the striatum, it is not possible to discern which region is responsible for the learning deficit. However, due to the highly restricted expression of *Gpr88*-Cre in MSNs, NMDARs in these cells appear to be essential for appetitive instrumental conditioning.

The inability of knockout mice to learn in the two-way active avoidance was particularly striking since these animals appear to respond normally to both sounds and shocks and since their locomotor activity in the chambers was intact. This finding is significant for a number of reasons. First, in addition to demonstrating a role for NMDARs in associating a tone with a foot shock, this experiment also demonstrates that animals lacking NMDARs in the striatum lack the ability to generate a behavioral response to prevent an adverse outcome. This result is in agreement with our finding that these knockout mice have a severe deficit in another form of cue-dependent learning, appetitive Pavlovian conditioning [36]. Second, it supports a growing body of evidence implicating the striatum in fear learning as well as appetitive learning. Specifically, excitotoxic or electrolytic lesions of the dorsal or ventral striatum have been shown to impair cued or contextual fear conditioning, respectively [37], [38]. However, these lesioning studies do not specifically implicate any class of neurotransmitters or receptors. Therefore, our results provide a direct demonstration that NMDARs in MSNs of the striatum are absolutely necessary for this type of learning.

Remarkably, removing *Grin1* from the MSNs of mice completely ablates their ability to learn all of the behaviors we tested. The severe learning deficits observed in knockout mice are reflected by their abnormal MSN morphology. MSN dendritic spine density was not examined here; however, the dramatic reduction of total dendrite length in the knockout mice is such that even if these animals had normal spine density and intact synaptic structure, the number of excitatory synapses onto MSNs of knockout animals would be reduced by approximately fifty percent. Because morphology was only assessed in adult animals, it is not clear whether MSNs fail to develop normally or atrophy as the animals age. However, other studies have implicated NMDAR signaling in normal neurite outgrowth and neuronal development [21]–[23]. Regardless of the precise etiology, altered MSN morphology in adult animals is compatible with normal daily activities but likely underlies their learning impairments.

## Methods

### Mice

All mouse lines used in these experiments were backcrossed to C57BL/6 mice for>10 generations. *Gpr88^CreGFP/+^* mice were generated by inserting a cassette encoding *CreGFP* fusion protein with a nuclear localization signal just upstream of the initiation codon in the second exon of *Gpr88* locus. Embryonic stem cells (G4) were electroporated, correctly targeted colonies were identified by Southern blot, and one of those colonies was injected into blastocysts to generate chimeras that were bred to produce mice carrying the *Gpr88^CreGFP^* allele. Mice with two conditional alleles for *Grin1* (*Grin1^lox/lox^* mice) [39] were crossed with *Mox2^Cre/+^* mice to generate mice with one globally inactivated *Grin1* allele (*Grin1^Δ/+^* mice). *Grin1^Δ/+^* mice were crossed to *Gpr88^CreGFP/+^* animals to generate *Gpr88* ^CreGFP/+^;*Grin1^Δ/+^* males. These males were crossed to *Gpr88^+/+^*;*Grin1^lox/lox^* females to generate *Gpr88^CreGFP/+^*;*Grin1^Δ /lox^* knockout mice and *Gpr88^CreGFP^* ^/*+*^;*Grin1^lox/+^* control mice. Male and female control and knockout mice were used in all experiments. No differences between males and females were observed in any of the behaviors tested; therefore, the data from both sexes were combined. All animals were between 8 and 10 weeks of age at the start of experiments. All animal protocols were approved by the University of Washington Institutional Animal Care and Use Committee.

### Immunohistochemistry

*Gpr88^Cre/+^* animals were sacrificed and perfused as described [40]. Briefly, animals were given a lethal dose of Beuthenasia and transcardially perfused with phosphate buffered saline (PBS) followed by 4% paraformaldehyde (PFA) in PBS. Brains were removed, postfixed in PFA overnight, cryoprotected in 30% sucrose, and frozen. Free-floating coronal sections (30 µm) were immunostained with a rabbit anti-GFP antibody (1∶1000, Invitrogen) and Cy2-conjugated secondary antibody (1∶200, Jackson ImmunoResearch), and were visualized using a Nikon Eclipse E600 microscope.

### Golgi staining

Portions of fixed striatum (∼0.2 cm^3^) were dissected and sectioned by vibratome in the coronal plane at 100 µm. Golgi–Cox staining was performed as described [41]. Using a Nikon 80i microscope (Melville, NY), well-impregnated MSNs were randomly selected for morphometric analysis from each slide by an observer blinded to genotype, according to described methods [42]. Morphometric measurements were made using Neurolucida (MicroBrightField, Williston, VT), as described by others [42].

### Western blots

Western blots were performed as described [29]. Briefly, mice were cervically dislocated and their striata and cortex were rapidly dissected on ice and frozen in liquid nitrogen. Tissue was sonicated in RIPA buffer (2.5% weight/volume) containing protease and phosphatase inhibitors, and centrifuged at 10,000 x g at 4°C for 10 min. Supernatant was collected and protein concentration assayed using a BCA assay (Thermo Scientific). Loading buffer was added to 25 µg total protein, samples were heated to 65°C for 15 min and electrophoresed on a pre-cast 4–20% gradient polyacrylamide gel (Bio-Rad). Protein was transferred onto nitrocellulose membranes and probed with anti-NR1 (1∶1000, Millipore) and anti- β-actin (1∶50000, Sigma) antibody. Membranes were then incubated with horseradish peroxidase-conjugated secondary antibodies and visualized (ECL, Amersham).

### Overnight locomotion

Animals were placed in locomotion chambers (Columbus Instruments) with *ad libitum* access to food and water for 48 hr. Distance traveled was calculated by Optomax software; the distance traveled during the second 12-hr light and dark cycles are reported to avoid the influence of novelty during the first 24 hr.

### Open field

Animals were placed in a circular open field measuring 50 cm in diameter for 10 min. The open field was divided into peripheral and a central 20-cm zone. Their activity was recorded by an overhead video camera and analyzed using Ethovision software. Total distance traveled, percent of time spent in the center versus periphery of the field, and number of center-crossings is reported.

### Rotarod

Mice were placed on an accelerating rod (Rotamex 4/8; Columbus Instruments) that increased in speed from 5 to 50 rpm over the course of a 5-min trial. Animals were given three trials a day, separated by 20 min, for three days. Latency to fall or to fail to stay on top of the rod is reported for each trial.

### Hanging wire grip test

The hanging-wire-grip test was performed as described [43]. Briefly, mice were placed on a 15.5-cm wide square of wire grid. The grid was gently raised and lowered three times, causing the animal to grip the grid. The grid was then quickly inverted and secured 42 cm above a padded surface. Latency to fall was measured, with a maximum trial time of 2 min. Each animal was given 3 trials with a 15-min intertrial interval. The average of the 3 trials was taken for each animal. Average latency to fall is reported for control and knockout animals.

### Operant conditioning

This paradigm was conducted in operant conditioning chambers (ENV-307W; Med Associates, Inc.) Mice were trained to retrieve food pellets in one 15-min magazine training session in which 10 food pellets (20 mg, BIO-SERV) were delivered randomly, on average every 90 s. Our criterion for inclusion in the operant conditioning experiment was successful retrieval of all food pellets during the magazine training session. One knockout and no controls were rejected on the basis of this criterion On subsequent days, mice underwent operant conditioning in which head entry into the food receptacle led to presentation of a lever. There were no predictive cues that signaled the start of a trial. One lever press led to delivery of a single food pellet, as well as retraction of the lever. The lever was presented again upon the next food receptacle head entry. Mice received one 60-min training session per day for seven consecutive days. For each session, the number of lever presses and number of head entries is reported.

### Two-way active avoidance

Two-way active avoidance was performed as described [30]. Briefly, mice were placed in a two-chamber active avoidance apparatus with free access to both chambers (PACS-30 two-way shuttle boxes, Columbus Instruments). After a 3-min habituation period animals began receiving trials in which a 7-s tone (80 dB, 2.5 kHz) overlapped with a 2-s foot shock (0.3 mA) presented during the last two seconds of the tone. Mice could avoid delivery of the foot shock by moving to the opposite side of the chamber during the first 5 seconds of tone presentation. Mice received 100 trials per day on four consecutive days. Each trial was followed by a 40-s intertrial interval (ITI). Throughout the training session the number of shuttles between chambers was recorded. In addition, the number of shock avoidances was recorded. Avoidance results were binned into blocks of 20 trials, and the percent of shocks avoided per block is reported. In addition, the number of shuttles between compartments per 100-trial training session is reported as ITI transfers.

### Acoustic startle

Acoustic startle was performed as described [44]. Animals were placed in sound-attenuating startle chambers (SR-Lab, San Diego Instruments). Following a 5-min habituation period in the chambers, animals were presented with a series of seven 40-ms tones with escalating sound levels ranging from a 0 dB (null trial) to 120 dB, with an ITI of 30 s between sound presentations. This series was presented 10 times in a 45-min session. The startle response was measured in 65 1-msbins starting at tone onset, and peak responses for each presentation at each dB level were averaged across the session.

### Shock reactivity

Naïve animals were placed in sound-attenuating startle chambers (SR-Lab, San Diego Instruments). Following a 5-min habituation period, animals were presented with ten 0.3-mA foot shocks, each lasting 0.5 sec with a 90-s ITI. Shock reactivity was measured in 500 1-ms bins beginning with the onset of shock presentation. Peak responses from each foot shock were averaged across the session.

### Statistics

Overnight locomotion, body weight, open field, hanging-wire-grip strength, and shock reactivity data were analyzed using unpaired t tests. Rotarod, operant conditioning, two-way active avoidance, startle response, and ITI transfer data were analyzed using two-way, repeated ANOVA. Fisher LSD post hoc tests were performed to assess within-group differences across days.
